# Editorial: Physiological ecology of trees under environmental stresses

**DOI:** 10.3389/fpls.2023.1158821

**Published:** 2023-02-16

**Authors:** Yang Liu, Songheng Jin, Guolei Li

**Affiliations:** ^1^ Jiyang College, Zhejiang A&F University, Zhuji, China; ^2^ School of Life Science and Health, Huzhou College, Huzhou, China; ^3^ College of Forestry, Beijing Forestry University, Beijing, China

**Keywords:** nutrient acquisition, environmental stresses, nitric oxide, functional traits, forest research, microbial diversity, molecular adaptation mechanisms, trees

The impact of environmental changes not only affects the survival of trees but also is closely related to the interests of human beings. Woody plants are a key element of the ecosystem since they help mitigate the negative impacts of complex climate changes such as increased carbon dioxide, high temperature, drought, etc (FAO, 2018). When trees suffer from environmental stress, they have to respond physiologically and ecologically to all kinds of adversity to improve their survival ability. This includes changes from population to individual, and involves the knowledge of ecology, morphology, and also physiology (Rewald et al., 2020). To explore the physiological ecology of trees under environmental stresses is of great significance to improve forestry production and environmental protection. The goal of this Research Topic (RT) is to present an overview of the fundamental discoveries in the field of physiological ecology of trees under environmental stresses. Here we collected physiological, biochemical, and also genetic studies to improve our understanding of the response mechanisms of trees under abiotic stresses, especially climate changes such as global warming, drought, atmospheric nitrogen deposition, salinization, etc.

In recent years, the occurrence of high temperature events has caused more scholars to study the heat tolerance of trees (Fahad et al., 2018; Yu, 2019). Ji et al. studied the effects of high temperature on photosynthesis between two tree peony varieties with different high temperature tolerance. Their results indicate the variety with strong high temperature tolerance had higher connectivity with reaction center of light capture complex, less damage to oxygen-evolving complex activity, and better stability of PSII system. Hu et al. combined physiological analyses and RNA seq technology to provide a holistic view of the behavior of *Betula luminifera* populations facing heat stress. Several transcription factors (TFs) genes were identified by differentially expressed genes analysis. Additionally, the author carried out qRT-PCR experiment to investigate the regulatory of candidate TFs under heat stress, which will lay the foundation for the selection and breeding of *Betula luminifera* and facilitate wider diversity of resistant *Betula luminifera* varieties to fulfill current and future needs.

Nutrient acquisition and high quality seedlings are the basis of successful afforestation (Davis and Jacobs, 2005). Thus, fertilization is often used to improve tree growth, and promote resistance to biotic and abiotic stresses (Zhu et al., 2020). However, the effect of fertilization depends on site conditions and external environments (Gessler et al., 2017). Song et al. reported nutrient internal cycles of two aged *Metasequoia* plantations in responses to N addition gradients and P addition gradients using two relative independent experiments. The authors identified the response patterns of root-soil accumulation factor (RSAF) and leaf nutrient resorption efficiency (LNRE) varied with fertilization types and stand ages, and explored the trade-off mechanism between RSAF and LNRE driven by nutrient alteration. Yu et al. compared the transcription level change of *Torreya grandis* using simulated N deposition and strigolactone (GR24) treatment. The authors analyzed the effects of these two treatments on the soil bacteria of *Torreya grandis*. The research showed 4,008 DEGs were identified and Legionella, Lacunisphaera, Klebsiella, Bryobacter, and Janthinobacterium were significantly enriched in the soil in the N addition and GR24 treatment. Yang et al. detected how different drought durations and N fertilization as well as their interactions affect the physiological and growth responses of two coexisting species saplings and their resilience abilities after rewetting procedure. They found root carbon storage appears to be extremely important for tree growth and survival under prolonged drought. Wang et al. conducted a summer drought and fertilization experiment, and analyzed how drought and fertilization induced changes in non-structural carbohydrate (NSC) storage of oak saplings affect its spring leaf development. Their results indicate that drought did not significantly alter NSC reserves, but delayed the spring leaf expansion and reduced the leaf biomass, while fertilization enhanced NSC reserves and stimulated spring leaf expansion.

Climate change is one of the main causes of soil salinization, which in recent years has become a growing problem in agriculture and leads to a decrease in the cultivation area, harming plant growth, crop yield, and quality (Deinlein et al., 2014; Sun et al., 2016; Shang et al., 2022). High salinity is often coupled with ionic and osmotic stress in trees, which alters the biochemical and physiological processes in the plants (Stefanov et al., 2022). Yang et al. discussed the role of nitric oxide in ameliorating salinity stress in dioecious plants and established fact that nitric oxide does mitigate salt-mediated oxidative injuries in a vast variety of crops and plants. Grafting and salt stress have been regarded as the main abiotic stress types for Chinese hickory. Yang et al. explained the role and expression analysis of PIN family genes in Chinese Hickory under salt stress and grafting. The authors had analyzed the expression of the PIN gene family. Their results showed CcPIN1a might be involved in the regulation of the grafting process, while CcPIN1a and CcPIN8a were related to the regulation of salt stress in Chinese hickory.

Light is an important ecological factor that affects plant growth, survival and distribution (Tang et al., 2015). Zhou et al. studied the effects of different light intensities on growth, physiology, photosynthetic characteristics, endogenous hormones and antioxidant activity responses of *Carpinus betulus* seedlings. The results suggested that *Carpinus betulus* can make effective use of low light resources by adjusting its morphology, material distribution, photosynthetic rate and antioxidant enzyme system in suitable low-light environments. Yang et al. conducted relative benefit and scaling relationship analyses of leaves in three urban plants to reveal the response and adaptation to different light conditions, which is helpful to understand the ecological changes in urban trees. Their results showed the leaves of the three urban plants exhibited a shift in strategy during transfer from the canopy shaded to the sunny habitat for adapting to the lower light conditions. Wu et al. conducted proteome analyses in developing *Styrax tonkinensis* kernels to revel the starch and oil biosynthesis under fruit shading treatment with four biological replicates. They found that fruit shading is a negative treatment for lipid accumulation but not starch accumulation by restraining enzymic protein expression involved in fatty acids (FAs) and triacylglycerol (TAG) biosynthesis during *Styrax tonkinensis* kernel development.

The invasion of moso bamboo to natural evergreen broad-leafed forests or mixed forests is a crucial issue of forest ecosystem and may cause important effect on forest ecological functions (Ramula and Pihlaja, 2012; Wang et al., 2019). In recent decades, moso bamboo has been largely increasing in the subtropical area of China, raising ecological concerns about its invasion into other native forest ecosystems (Wang et al., 2017). While the ecological impacts of moso bamboo invasion into native forests have been widely studied, no study has considered its impact on forest productivity and on the relationship between species diversity and productivity. Chen et al. supplied reliable results on the impacts of bamboo invasion on species diversity and biomass. The authors found that bamboos exchange water *via* rhizomes and nighttime fluxes are highly important for the support of freshly sprouted culms.

In recent years, with the rapid development of industrialization and urbanization, the accumulation of heavy metals (HMs) in the environment has been increasing, especially in developed economic regions (Yang et al., 2020). As a developed economic region in China, the problem of HMs pollution in the Yangtze River Delta has become increasingly prominent. Li et al. illustrated a human health risk assessment of heavy metals in the bark and leaves of camphor trees located in Yangtze River Delta, China. The source and contribution rate of heavy metals in the study area were analyzed by Pb isotope ratio, and the human health risk assessment model was combined with Pb isotope ratio analysis to evaluate the health risk of heavy metal pollution sources in the study area. Their research results can provide scientific guidance for the prevention and control of heavy metal pollution of camphor trees and provide a reference for avoiding areas with high risk of heavy metal pollution.

## Author contributions

YL and SJ provided the first draft of the MS. All co-authors jointly revised the manuscript and approved its publication.
